# Adverse drug events observed with 150 mg versus 300 mg secukinumab for the treatment of moderate to severe plaque psoriasis: A systematic review and meta-analysis

**DOI:** 10.1097/MD.0000000000014042

**Published:** 2019-01-11

**Authors:** Li Zhang, Hua Yang, Qihong Chen, Jing Zhao

**Affiliations:** a Department of Dermatology; b Department of Cardiology, Jingzhou Central Hospital, The Second Clinical Medical College, Yangtze University, Jingzhou, Hubei, China.

**Keywords:** adverse drug events, drug discontinuation, Psoriasis, secukinumab

## Abstract

**Background:**

Secukinumab has been approved for the treatment of moderate to severe plaque psoriasis. However, safety measures concerning drug administration is vital during treatment. Understanding the right way to administer drugs is important to reduce any serious adverse drug event. In this analysis we aimed to systematically show the risk of adverse drug events which were observed with 150 mg versus (vs) 300 mg secukinumab for the treatment of moderate to severe plaque psoriasis.

**Methods:**

The major online databases: Cochrane Central, MEDLINE, www.ClinicalTrials.com and EMBASE were searched for relevant publications based on the comparison of secukinumab 150 mg vs 300 mg for the treatment of moderate to severe plaque psoriasis. Adverse drug events were considered as the clinical endpoints. Statistical analysis was carried out by the RevMan 5.3 software. Risk ratios (RR) and 95% confidence intervals (CIs) were generated to represent the data following statistical analysis.

**Results:**

Seven studies with a total number of 2361 participants were included. Results of this analysis showed that the risk of any adverse event (RR: 1.00, 95% CI: 0.96–1.05; *P* = .94), the risk of serious adverse events (RR: 1.04, 95% CI: 0.75–1.43; *P* = .82) and the risk of adverse events leading to drug discontinuation (RR: 0.98, 95% CI: 0.61–1.57; *P* = .92) were not significantly different between 150 mg vs 300 mg secukinumab for the treatment of moderate to severe plaque psoriasis. When the detailed adverse drug events were studied, the risks of infection or infestation (RR: 1.11, 95% CI: 0.98–1.25; *P* = .09), naso-pharyngitis (RR: 1.05, 95% CI: 0.90–1.23; *P* = .55), headache (RR: 0.92, 95% CI: 0.68–1.25; *P* = .60), diarrhea (RR: 1.14, 95% CI: 0.75–1.73; *P* = .55), pruritus (RR: 0.82, 95% CI: 0.56–1.22; *P* = .33), arthralgia (RR: 0.96, 95% CI: 0.67–1.38; *P* = .83), upper respiratory tract infection (RR: 0.98, 95% CI: 0.70–1.36; *P* = .89), hypertension (RR: 1.22, 95% CI: 0.83–1.81; *P* = .31), nausea (RR: 1.39, 95% CI: 0.63–3.04; *P* = .42), and cough (RR: 1.46, 95% CI: 0.67–3.19; *P* = .34) were still not significantly different between these 2 dosage regimens.

**Conclusion:**

Secukinumab 150 mg and 300 mg were both equally tolerable and might safely be used for the treatment of moderate to severe plaque psoriasis. No significant adverse drug events were observed with any of the dosage.

## 1 Introduction

Psoriasis which is an autoimmune disorder of the skin, has reached more than seven million cases in the United States.^[1]^ This chronic disease is often characterized by red, itchy, dry, and scaly skin patches. There are several types of psoriasis, however, plaque psoriasis is the most common comprising of up to 90% of cases.^[2]^

Plaque psoriasis is characterized by raised red patches covered with a whitish build-up of dead skin and it mainly appears on the elbow, knees, scalp, and lower back, but it might also be present in the other surfaces of the body.

To understand the treatment of psoriasis, it is necessary to have a knowledge of the pathogenesis involved in the development of this chronic disease. In brief, the mechanism of this autoimmune disease is based on the expression of the cytokine interleukin 17A.^[3]^

Secukinumab (Cosentyx), a human IgG1k monoclonal antibody, binds and inhibits/neutralizes interleukin 17A and this is how it is involved in the treatment of psoriasis.^[4]^ This novel drug was approved by the Food and Drug Administration in January 2015 for the treatment of adults with moderate to severe plaque psoriasis and was prescribed as secukinumab 150 mg and 300 mgrespectively.^[5]^

Several recent research showed secukinumab to be effective in the treatment of moderate to severe plaque psoriasis.^[6]^ However, the dosage to be used was often a question to both the physicians and the patients.

Safety measures concerning drug administration is vital during the treatment of patients with specific drugs.^[7]^ Drugs might be harmful even if they are meant to improve the health of a patient and taking them correctly and understanding the right way to administer them is important to reduce any serious adverse drug event.

In this analysis we aimed to systematically show the risk of adverse drug events which were observed with 150 mg versus (vs) 300 mg secukinumab for the treatment of moderate to severe plaque psoriasis.

## 2 Methods

### 2.1 Search databases and search strategies

The major online databases: Cochrane Central, MEDLINE, www.ClinicalTrials.com and EMBASE were searched for relevant publications based on the comparison of secukinumab 150 mg vs 300 mg for the treatment of moderate to severe plaque psoriasis. The time frame for retrieval of articles was from the beginning of August to the end of September 2018.

Only English publications were considered for this research article and the search process was carried out using the following search terms:

- Secukinumab and psoriasis;
- Secukinumab and plaque psoriasis;
- Secukinumab and moderate to severe psoriasis;
- Secukinumab, psoriasis and adverse drug events.

### 2.2 Inclusion and exclusion criteria

Inclusion criteria was based on studies which:

- Were randomized trials or observational studies;
- Compared secukinumab 150 mg vs 300 mg for the treatment of moderate to severe plaque psoriasis;
- Reported adverse drug events as their clinical endpoints;
- Reported data which could be used in this meta-analysis.

Exclusion criteria was based on studies which:

- There were not randomized trials and observational cohorts;
- Did not compare secukinumab 150 vs 300 mg for the treatment of psoriasis;
- Did not report the relevant adverse drug events;
- Reported data which were not compatible with this analysis;
- Were duplicated studies.

### 2.3 Endpoints (adverse drug events), types of participants and follow-up time periods

Most of the participants were patients who were treated for moderate to severe plaque psoriasis as shown in Table 1.

**Table 1:**
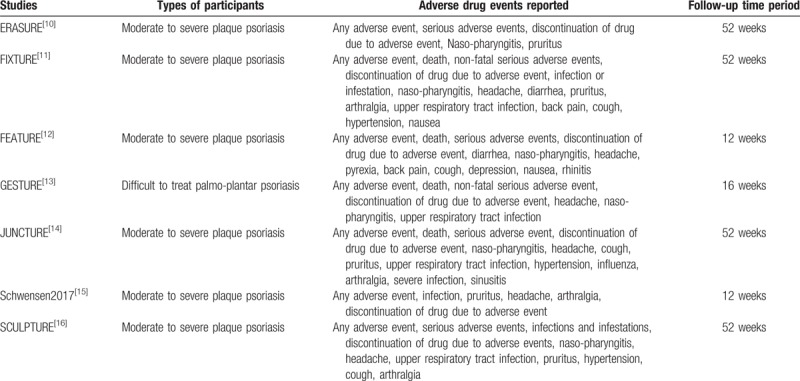
Types of participants, adverse drug events reported and follow-up time period.

The adverse drug events which were assessed included:

1. Any adverse event;
2. Serious adverse events;
3. Adverse events leading to drug discontinuation;
4. Infection or infestation;
5. Naso-pharyngitis;
6. Headache;
7. Pruritus;
8. Arthralgia;
9. Upper respiratory tract infection
10. Hypertension;
11. Nausea;
12. Cough.

Four studies reported a follow-up time period of 52 weeks, 2 studies reported a follow-up time period of 12 weeks and one study reported a follow-up time period of 16 weeks as shown in Table 1.

### 2.4 Data extraction, quality assessment, and statistical analysis

The original studies were reviewed and the following data were extracted by the respective four authors (LZ, HY, QC, JZ):

1. Total number of participants assigned to 150 vs 300 mg secukinumab respectively;
2. The endpoints reported, with respective follow-up time periods;
3. The baseline characteristics of the participants;
4. The main features of the original articles;
5. The total number of adverse events in each category of subgroup.

The methodological quality of the trials was assessed with reference to the recommendations from the Cochrane collaboration^[8]^ whereby bias risk was labelled as ‘low risk’, ‘moderate risk’ and ‘high risk’.

Any disagreement which followed was completely resolved by consensus.

Statistical analysis was carried out by the RevMan 5.3 software. Risk ratios (RR) and 95% confidence intervals (CIs) were generated to represent the data following statistical analysis.

Heterogeneity was assessed by the Q statistic test whereby a result with a *P* value less or equal to .05 was considered as statistically significant, and a result reporting a *P* value above .05 was considered statistically insignificant.

Heterogeneity was also assessed by the I^2^ statistic test. The I^2^ value was represented by percentage. A higher percentage of I^2^ denoted a higher heterogeneity whereas a lower percentage denoted a lower heterogeneity.

In addition, a fixed statistical model was used if the I^2^ value was less than 50%, whereas a random statistical model was used if the I^2^ value was more than 50%.

Sensitivity analysis was carried out by a method of exclusion. Each study was excluded at a time and a new analysis was generated to observe for any significant difference from the main results.

Publication bias was visually observed by assessing funnel plots.

### 2.5 Ethical approval

Ethical approval or board review approval was not required for this study.

## 3 Results

### 3.1 Search outcomes

Following the PRISMA guideline,^[9]^ a total number of 675 publications were retrieved from online databases. Following a careful assessment of the titles and abstracts, 596 articles were eliminated due to irrelevance.

Seventy-nine (79) full text articles were assessed for eligibility.

Following further assessments, other full text publications were eliminated due to the following reasons:

- Literature review (2);
- Meta-analysis and pooled studies (7);
- Did not report relevant endpoints (6);
- Control group was absent (7);
- Did not report the relevant dosage of drug (2);
- Included data which could not be used (3);
- Duplicated studies (45).

**Figure 1:**
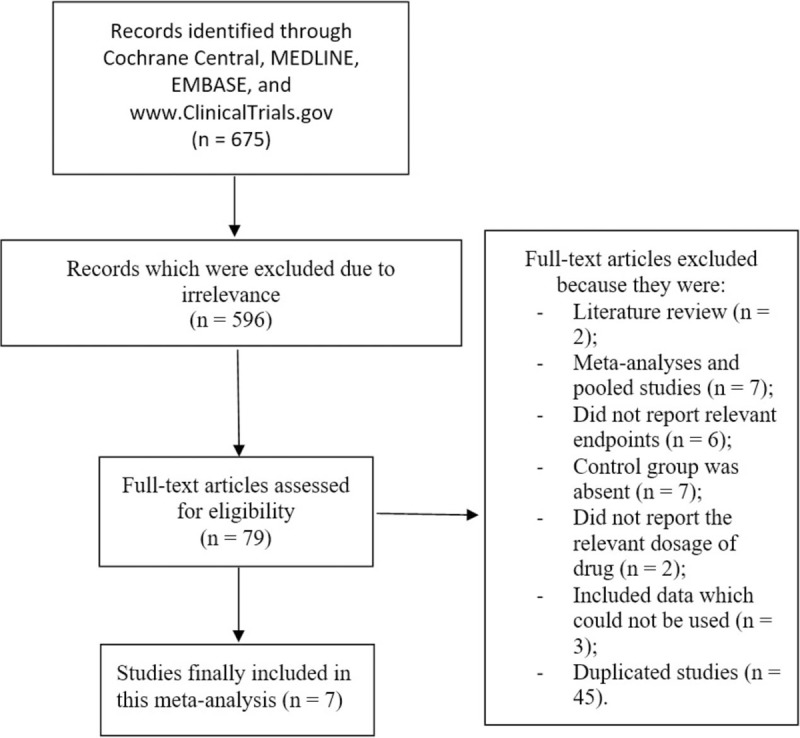
Flow diagram representing the selection of studies for this analysis.

Finally, only 7 studies^[10–16]^ were included in this meta-analysis. The flow diagram for the study selection has been demonstrated in Figure 1.

### 3.2 Main and baseline features of the studies and participants respectively

The main features of the original studies were listed in Table 2.

**Table 2:**
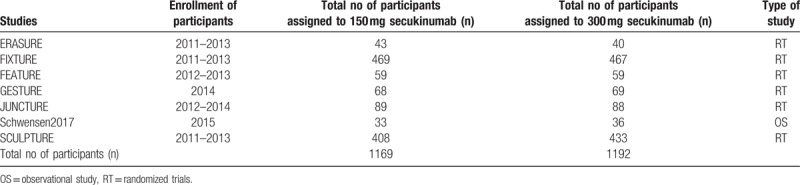
Main features of the studies.

A total number of 2361 participants were included in this meta-analysis comparing 150 mg vs 300 mg secukinumab for the treatment of moderate to severe plaque psoriasis. The 1169 participants were assigned to 150 mg secukinumab whereas 1192 participants were assigned to 300 mg secukinumab as shown in Table 2.

Six studies were randomized trials and 1 study was an observational cohort.

The enrollment time period of the participants varied from year 2011 to 2015.

The baseline characteristics of the participants were listed in Table 3. Mean age of the participants ranged from 43.9 to 52.4 years. Male participants (51.1–72.2) % were pre dominant in comparison to female participants. The body mass index (BMI) varied from 28.4 to 30.6 kg/m^2^. A minor population of the participants also suffered from psoriatic arthritis. Duration of psoriasis varied from 7.5 to 20.4 years as shown in Table 3.

**Table 3:**
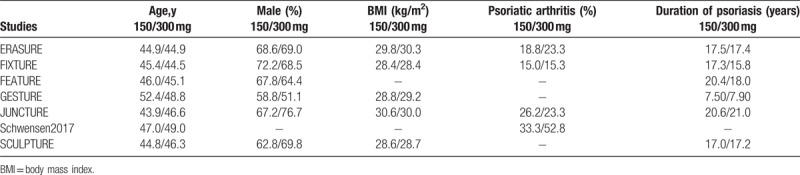
Baseline features of the participants.

### 3.3 Comparing the adverse drug events observed with 150 mg vs 300 mg secukinumab for the treatment of moderate to severe plaque psoriasis

**Figure 2:**
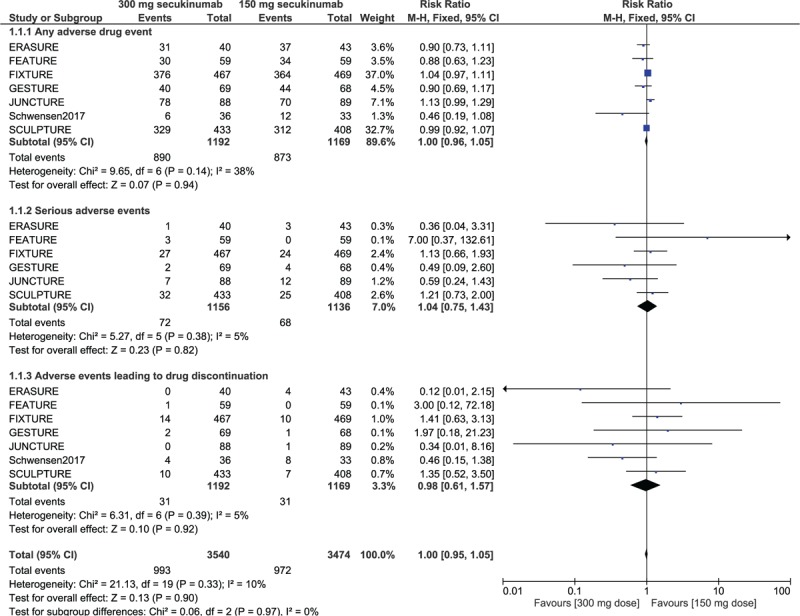
Adverse drug events observed with 150 mg vs 300 mg secukinumab for the treatment of moderate to severe plaque psoriasis.

Results of this analysis showed that the risk of any adverse event (RR: 1.00, 95% CI: 0.96–1.05; *P* = .94), the risk of serious adverse events (RR: 1.04, 95% CI: 0.75–143; *P* = .82) and the risk of adverse events leading to drug discontinuation (RR: 0.98, 95% CI: 0.61–1.57; *P* = .92) were not significantly different between 150 mg vs 300 mg secukinumab for the treatment of moderate to severe plaque psoriasis as shown in Figure 2.

**Figure 3:**
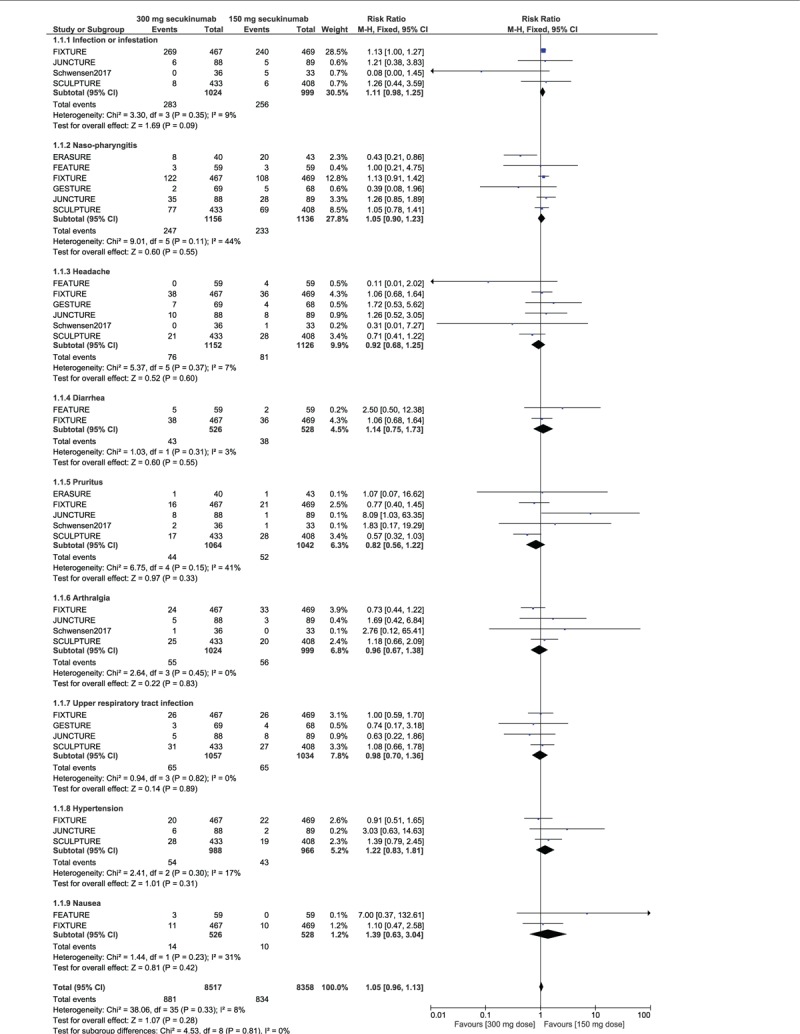
Detailed adverse drug events observed with 150 mg vs 300 mg secukinumab for the treatment of moderate to severe plaque psoriasis.

**Figure 4:**
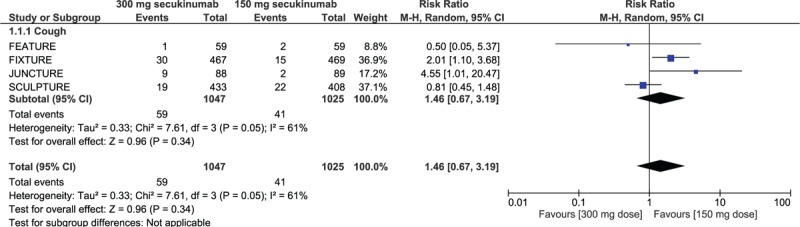
Cough as an adverse drug event observed with 150 mg vs 300 mg secukinumab for the treatment of moderate to severe plaque psoriasis.

When the adverse drug events were studied in details, the risks of infection or infestation (RR: 1.11, 95% CI: 0.98–1.25; *P* = .09), naso-pharyngitis (RR: 1.05, 95% CI: 0.90–1.23; *P* = .55), headache (RR: 0.92, 95% CI: 0.68–1.25; *P* = .60), diarrhea (RR: 1.14, 95% CI: 0.75–1.73; *P* = .55), pruritus (RR: 0.82, 95% CI: 0.56–1.22; *P* = .33), arthralgia (RR: 0.96, 95% CI: 0.67–1.38; *P* = .83), upper respiratory tract infection (RR: 0.98, 95% CI: 0.70–1.36; *P* = .89), hypertension (RR: 1.22, 95% CI: 0.83–1.81; *P* = .31), nausea (RR: 1.39, 95% CI: 0.63–3.04; *P* = .42), and cough (RR: 1.46, 95% CI: 0.67–3.19; *P* = .34) were still not significantly different between 150 mg vs 300 mg secukinumab for the treatment of moderate to severe plaque psoriasis as shown in Figures 3 and 4.

A summarized version of the results has been listed in Table 4.

**Table 4:**
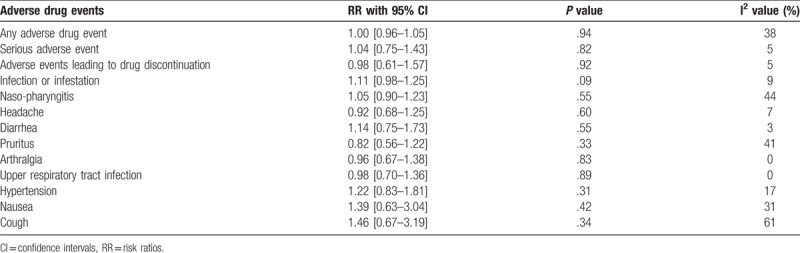
Results of this analysis.

**Figure 5:**
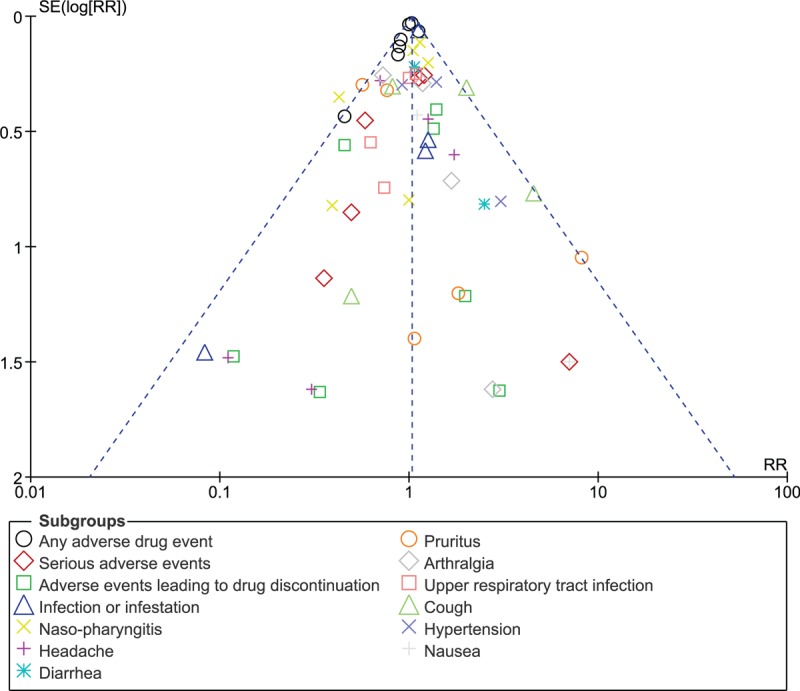
Funnel plot showing publication bias.

Sensitivity analysis resulted in consistent results throughout. Additionally, a low evidence of publication bias was observed across all the studies that assessed the adverse drug events observed between the two different dosages of secukinumab for the treatment of patients with moderate to severe plaque psoriasis as shown in Figure 5.

## 4 Discussion

Our results showed that both dosages of secukinumab were equally safe to be used for the treatment of moderate to severe plaque psoriasis. There was no significant difference in adverse drug events observed with any of the dosage.

Similar to the results of this analysis, in order to assess the long-term safety of secukinumab, a pooled analysis of 10 phase II and III clinical studies in patients with moderate to severe plaque psoriasis showed comparable adverse drug events during this 52 week follow-up time period.^[17]^ However, in this current analysis, we only compared secukinumab 150 mg vs 300 mg, and we did not include phase II trials.

Results from 2 randomized phase 3 trials showed secukinumab to significantly improve physical function in participants with plaque psoriasis and psoriatic arthritis.^[18]^ In addition, it was observed that physical functioning as well as the condition of this chronic disease were more significantly improved with secukinumab 300 mg.

Even in the JUNCTURE trial, the authors concluded that secukinumab was effective, well-tolerated and was associated with high usability for the treatment of moderate to severe psoriasis.^[19]^ The fact that responses were much better with secukinumab 300 mg should not be ignored. Better response with tolerable safety drug events might further guide therapies in patients with moderate to severe plaque psoriasis. The drug was also well accepted in North Americans.^[20]^

Our research aimed to compare the adverse drug events observed with secukinumab 150 mg vs 300 mg for the treatment of moderate to severe plaque psoriasis and the results showed both dosages to be well-tolerated without any significantly higher adverse event. Other research have shown secukinumab to be very effective, especially the 300 mg dosage, for the treatment of moderate to severe plaque psoriasis. Therefore, secukinumab 300 mg might be considered by physicians for the treatment of this chronic autoimmune condition. However, future phase IV trials should further assess this interesting drug.

### 4.1 Limitations

The limitations were as followed: The total number of participants was limited in comparison to other studies. Secondly, we also included one study with participants who were being treated for palmo-plantar psoriasis in comparison to all the other original studies which included patients with moderate to severe plaque psoriasis. In addition, most of the follow-up time periods reported were 52 weeks, however, one study had a follow-up time period of 16 weeks and 2 other studies had a follow-up time period of 12 weeks. Also, whether other drugs were being alternatively used were not reported or taken into consideration in this analysis and this might have influenced the endpoints.

## 5 Conclusion

Secukinumab 150 mg and 300 mg were both equally tolerable and might safely be used for the treatment of moderate to severe plaque psoriasis. No significant adverse drug events were observed with any of the dosage.

## Acknowledgments

All named authors meet the International Committee of Medical Journal Editors (ICMJE) criteria for authorship for this article, take responsibility for the integrity of the work as a whole, and have given their approval for this version to be published.

## Author contributions

LZ, HY, QC and JZ were responsible for the conception and design, acquisition of data, analysis and interpretation of data, drafting the initial manuscript and revising it critically for important intellectual content.

**Conceptualization:** Li Zhang, Hua Yang, Qihong Chen, Jing Zhao.

**Data curation:** Li Zhang, Hua Yang, Qihong Chen, Jing Zhao.

**Formal analysis:** Li Zhang, Hua Yang, Qihong Chen, Jing Zhao.

**Funding acquisition:** Li Zhang, Hua Yang, Qihong Chen, Jing Zhao.

**Investigation:** Li Zhang, Hua Yang, Qihong Chen, Jing Zhao.

**Methodology:** Li Zhang, Hua Yang, Qihong Chen, Jing Zhao.

**Project administration:** Li Zhang, Hua Yang, Qihong Chen, Jing Zhao.

**Resources:** Li Zhang, Hua Yang, Qihong Chen, Jing Zhao.

**Software:** Li Zhang, Hua Yang, Qihong Chen, Jing Zhao.

**Supervision:** Li Zhang, Hua Yang, Qihong Chen, Jing Zhao.

**Validation:** Li Zhang, Hua Yang, Qihong Chen, Jing Zhao.

**Visualization:** Li Zhang, Hua Yang, Qihong Chen, Jing Zhao.

**Writing – original draft:** Li Zhang, Hua Yang.

**Writing – review & editing:** Li Zhang, Hua Yang.
